# Comparison of microbiota in the upper versus lower respiratory tract in children during health and respiratory disease: protocol for a systematic review

**DOI:** 10.1186/s13643-021-01806-2

**Published:** 2021-09-21

**Authors:** Richa Rao, Jeanne M. Dsouza, Joseph L. Mathew

**Affiliations:** 1 Department of Pediatrics, Postgraduate Institute of Medical Education and Research, Chandigarh, 160012 India; 2 Kasturba Medical College, Manipal University, Manipal, 576104 India

**Keywords:** Micro-organism, Microbial flora, Comparison, Lungs, Nasopharyngeal, Oropharyngeal, Respiratory tract, Pneumonia

## Abstract

**Background:**

The upper respiratory tract of children is colonized by various microbial species during the healthy state, whereas the lungs are believed to be sterile. In children with respiratory infections, micro-organisms can be recovered from the upper respiratory sites, as well as the lungs. However, the correlation of microbial yield between the two sites is unclear. This systematic review is designed to explore the microbial composition of the respiratory system in healthy children, comparing the organisms identified in the upper airways versus the lungs. We will also compare the prevalence and pattern of upper respiratory micro-organisms in healthy children versus those with various respiratory diseases. We will additionally compare the organisms identified in the upper airway versus the lungs in children with respiratory disease.

**Methods:**

We will search the following electronic databases: Epistemonikos and Cochrane Library for systematic reviews and MEDLINE (through PubMed), EMBASE, Cochrane CENTRAL, LIVIVO, Web of Science, Scopus, and CINAHL databases for primary studies. Reference lists of relevant studies will be examined for links to potential related articles. Two reviewers will independently determine eligibility for inclusion. The methodological quality and risk of bias of the included observational studies will be scored using the Newcastle–Ottawa Scale tool, and JBI Critical Appraisal Checklist for case series. We will present the data with descriptive statistics and provide pooled estimates of outcomes, wherever it is feasible to perform a meta-analysis. Heterogeneity in studies will be explored by using the Higgins and Thompson *I*^2^ method. Sensitivity analysis will be done to explore the impact of study quality, and subgroup analysis will be done based on age, health condition, type of respiratory specimen, and method of identifying organisms. We will prepare a summary of findings’ table and assess the confidence in the evidence using the GRADE methodology.

**Results:**

This is a protocol; hence, there are no results at this stage.

**Discussion:**

The proposed systematic review will provide comparisons of the microbiota in the upper respiratory tract versus the lungs, in children, during health as well as respiratory disease. Similarly, the site-specific yield will be compared between healthy children and those with respiratory disease. This will provide clinicians, microbiologists, and respiratory therapists a better understanding of the respiratory system microbiota, suitability (or otherwise) of upper airway specimens in various respiratory diseases, and the potential role of upper airway colonization on specific respiratory diseases. We will disseminate the review through a peer-reviewed journal publication. Data that cannot be included in the published version will be made available on request.

**Systematic review registration:**

PROSPERO CRD42020202115.

## Background and rationale

The term “microbiota” refers to the various species of micro-organisms that live in a defined environment. In the human body, microbiota are present in organs that are in contact with the outside environment [1]. Microbiota may differ among various individuals and also within an individual during health and disease states. The term “microbiome” includes the complete set of micro-organisms (bacteria, viruses, and fungi) with their genomes [2]. There are numerous mutually beneficial interactions between the human body and microbiota, which are important for maintaining health. Microbiota can also contribute to the pathogenesis of some diseases [3]. Until recently, the microbial structure of the human body was poorly understood. However, large-scale research conducted within the framework of the Human Microbiome Project (HMP 2007) enhanced knowledge on the diversity of the microflora in human beings [4].

The upper respiratory tract is colonized by a variety of different microbial species right after birth. The initial colonization depends on the mode of delivery (vaginal or cesarean section) [5]. Dramatic changes occur during the first year of life, probably driven by the maturation of the immune system and dietary practices [6]. The microbial community in infants and children gradually transforms into the adult upper respiratory tract microbiome, becoming less dense but more diverse [7]. There are differences in the composition of the upper respiratory tract microbiome between healthy volunteers and those suffering from various respiratory diseases [8].

Previously, the lower respiratory tract was assumed to be sterile, except during infection. This concept existed due to limited experimental access to the lower respiratory tract of healthy individuals and limitations of classical methods of culturing organisms [9]. Therefore, the study of the lungs was not even included in the original Human Microbiome Project. However, recent studies, using culture-independent techniques, demonstrated the presence of *Actinobacteria*, *Proteobacteria*, *Bacteroidetes*, and *Firmicutes* ribosomal DNA in the lungs of healthy individuals [10]. Bacteria have been identified using more sensitive techniques, especially the 16S rRNA gene [11]. Recent studies using next-generation sequencing (NGS) have enabled accurate identification of bacterial genetic material in the lungs of healthy children also [8, 12].

Sampling of the upper airway at any age (using nasal swabs or nasopharyngeal aspirates or nasopharyngeal swabs or orophrayngeal swabs) is fairly straightforward [13]. The inaccessibility of the lungs poses significant challenges for researchers. Sampling from the lungs and distal airways in adults is generally done using sputum or endoscopic bronchoalveolar lavage [14]. However, this is neither easy nor reliable in the pediatric age group, as infants and young children often do not expectorate sputum, specimen collection is challenging, and invasive procedures are rarely used for reliable specimen collection. Therefore, many clinicians resort to using upper airway specimens as surrogates for lower airway or lung specimens. Common examples are nasopharyngeal swabs or aspirates to determine pneumonia etiology [15, 16], and throat or oropharyngeal swabs in children with cystic fibrosis [17, 18]. However, the reliability of this as surrogates for lung specimens has not been clearly defined in various respiratory diseases.

Currently, there is no comprehensive review of evidence comparing the prevalence and pattern of microbiota identified in the upper respiratory tract versus the lungs of children with respiratory disease. There is also paucity of evidence comparing the upper airway microbiota of healthy children versus those with respiratory disease. This systematic review is being conducted to address these gaps in the literature.

### Objectives

The overall objective of this systematic review of literature is to compare the microbial flora of the upper respiratory tract versus the lungs, among infants and children, during the healthy state as well as episodes of respiratory disease. An additional objective is to compare the pattern of upper airway colonization in healthy children versus those with respiratory disease. These objectives will be addressed through the following specific review questions.

Review questions:

1. What are the microbial organisms present in the upper airway respiratory specimens of healthy children?
2. What are the microbial organisms present in the lungs of healthy children?
3. In healthy children, what is the comparison between the microbial flora present in the upper airway respiratory specimens versus the lungs?
4. What is the comparison between the upper airway microbial flora in healthy children versus children with various respiratory diseases?
5. What is the comparison between the lung microbial flora in healthy children versus children with various respiratory diseases?
6. In children with various respiratory diseases, what is the comparison between the microbial flora present in the upper airway respiratory specimens versus the lung?

## Methods

**Fig. 1:**
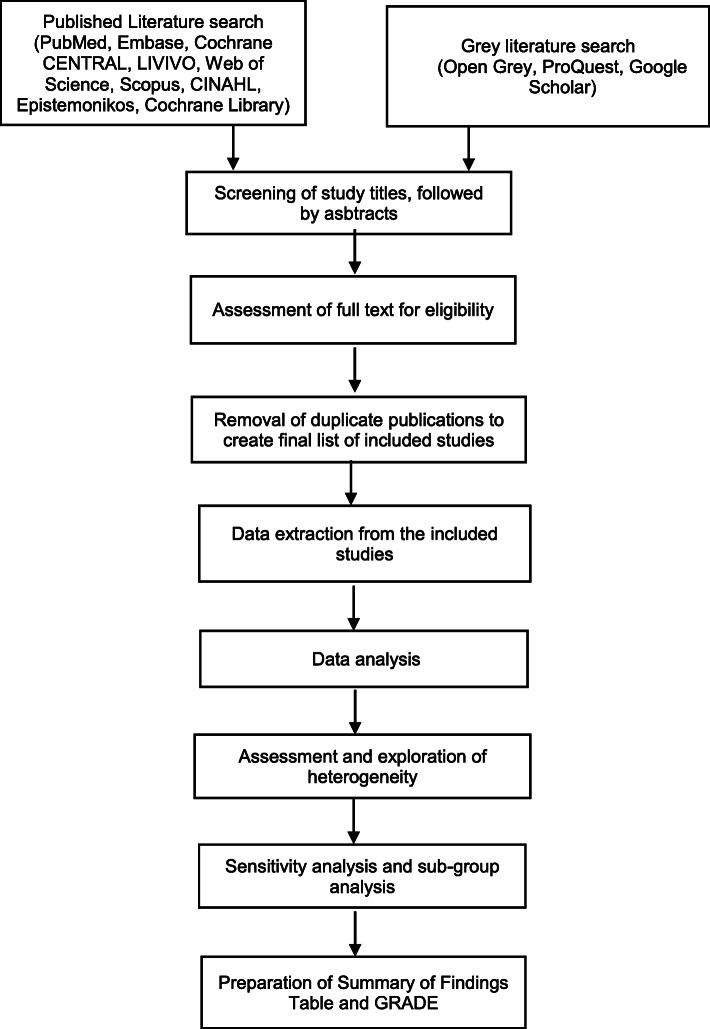
Flowchart of the systematic review

This protocol has been registered within the PROSPERO database (CRD42020202115). This review will follow the relevant domains of the PRISMA-P (Preferred Reporting Items for Systematic Reviews and Meta-Analyses-Protocols) statement for quantitative aspects. Figure 1 summarizes the flow of the systematic review process.

### Eligibility criteria

#### Types of studies

We will include all study designs that have the potential to address one or more of the review questions outlined above. These include observational studies, notably prospective cohort studies, retrospective cohort studies, case–control studies, and case series. Data may be available from one or other arms of randomized and non-randomized clinical trials as well; hence, these will be included also. We will also include any existing systematic reviews of literature that address any of the review questions.

We will exclude narrative reviews that do not provide objective data and also case series with less than 5 participants. We will also exclude studies related to animal experiments, in vitro experiments, and ex vivo human studies.

Broadly, we expect to include the following types of studies:

1. Those which describe micro-organisms in the upper airway respiratory tract and/or lungs of healthy children
2. Those which report micro-organisms in the upper airway respiratory tract and simultaneously the lungs of children with respiratory diseases
3. Those which compare micro-organisms in the upper airway respiratory tract of healthy children versus those with respiratory diseases
4. Those which compare micro-organisms in the lungs of healthy children versus those with respiratory diseases

#### Types of participants

We will include studies conducted in children (age range birth to 18 years) who are normal or healthy or asymptomatic (as defined by authors of individual publications) as well as those with acute or chronic respiratory disease (as defined by the authors of publications).

We will exclude studies that report findings in participants older than 18 years of age, or where data of children and adults are presented together, without the possibility of extracting data of children separately. We will also exclude post-mortem studies. If potentially eligible studies report data from specimens such as tracheal aspirate, tracheostomy tube secretions, and endotracheal tube aspirates, these will be excluded (as it is difficult to clearly classify them as upper or lower respiratory specimens).

#### Types of exposure

We will include studies reporting the microbiota identified in children during health and respiratory disease, irrespective of the age of the children, timing of specimen collection, methods used for specimen collection, and microbiologic processing technique. Since this is not a systematic review of interventions, no specific interventions will be considered. However, if studies addressing one or more of the review questions include any intervention(s), these will be not be excluded.

### Types of outcome measures

1. Organisms (bacteria, viruses, fungi) identified in upper respiratory tract specimens of healthy children
2. Organisms (bacteria, viruses, fungi) identified in the lungs of healthy children
3. Organisms (bacteria, viruses, fungi) identified in upper respiratory tract specimens and simultaneous lung specimens of children with respiratory diseases

### Information sources

Two authors will independently search the following electronic databases to identify relevant studies: Epistemonikos and Cochrane Library for systematic reviews and MEDLINE (through PubMed), EMBASE, Cochrane CENTRAL, LIVIVO, Web of Science, Scopus, and CINAHL databases for primary studies. All searches will be run from inception to 30 June 2021, or the actual date of publication of the protocol, whichever is later. There will be no restrictions based on language or geographies.

### Handsearching

The authors will check reference lists of all primary studies and review articles for additional references. Articles identified through reference lists and bibliographic searches will also be considered for data extraction.

### Grey literature

We will conduct a grey literature search (to identify studies not indexed in the databases listed above) using OpenGrey (www.opengrey.eu/), ProQuest, and Google Scholar.

### Search strategy

MeSH terms and synonyms for the following keywords will be combined together to run the literature search: “child, micro-organism, respiratory.” A typical search string based on this is as follows: “(organism OR microorganism OR micro-organism OR micro organism OR microbe OR bacteria OR virus OR fungus OR microbiome OR microbiota) AND (child OR pediatr*) AND (respiratory OR airway OR lung OR nasopharyngeal OR oropharyngeal OR nasal OR throat).” Pilot testing of this search string yielded over 67,000 citations in PubMed, confirming that the search strategy is comprehensive and unlikely to miss any relevant citations.

We will use the Peer Review of Electronic Search Strategies (PRESS) checklist for systematic reviews, for structured reviews of our literature search strategies [19]. The checklist is designed to identify errors in the search strategy and enhance the search.

### Study records

#### Selection process

Two review authors will independently screen titles followed by abstracts of all studies identified through the searches and then retrieve the full-text study reports/publications. Two authors will independently screen the full text and identify studies for inclusion. They will also identify and record reasons for exclusion of the ineligible studies. A table will be presented listing the studies excluded from our synthesis at the full-text stage and the reasons for exclusion.

Any disagreements will be discussed and resolved among review authors, with arbitration by an external expert if necessary. Where the same study, using the same sample and methods, is presented in different reports, such reports will be collated so that each study, rather than each report, is the unit of interest in the review. After removal of duplicate publications, the final list of included studies will be created. The study screening form as well as the data extraction form to be used in this systematic review will be pilot tested in advance to ensure there are no errors. A PRISMA flow diagram will be used to illustrate the search results and the process of screening and selecting studies for inclusion.

### Data management

We will use Rayyan (https://www.rayyan.ai) for the management of the screening and data extraction stages of the systematic review.

### Translation of publications in languages other than English

For papers that are published in a language other than English, the abstract will be subject to initial translation through open source software. If this indicates potential inclusion, or if the translation is inadequate to permit a decision, an attempt will be made to obtain a formal translation of the full text. If this cannot be done, the authors will categorize the study as “awaiting classification” to ensure transparency in the review process.

### Data collection process (data extraction and management)

#### Dealing with missing data

We will contact the corresponding authors of studies where data is/are missing and try to obtain the missing data. If this fails, we will try and impute data where possible. If that is not feasible, we will state as such.

#### Outcomes and prioritization

1. Organisms (bacteria, viruses, fungi) identified in upper respiratory tract specimens of healthy children, with the frequency of each organism identified and proportion of children with each organism type
2. Organisms (bacteria, viruses, fungi) identified in the lungs of healthy children, with the frequency of each organism identified and proportion of children with each organism type
3. Comparison of organisms (bacteria, viruses, fungi) identified in upper respiratory tract specimens of healthy children versus organisms identified in their lungs, with the frequency of each organism identified and proportion of children (in each group) with each organism type
4. Organisms (bacteria, viruses, fungi) identified in upper respiratory tract specimens and simultaneous lung specimens of children with respiratory diseases, with the frequency of each organism identified and proportion of children with each organism type (in both sites)
5. Comparison of organisms identified in upper respiratory tract specimens of healthy children versus those with respiratory diseases, with the frequency of each organism identified and proportion of children (in both groups) with each organism type
6. Comparison of organisms identified in lungs of healthy children versus those with respiratory diseases, with the frequency of each organism identified and proportion of children (in both groups) with each organism type

### Data synthesis

The data obtained will be described in detail. We will try to pool data and perform meta-analysis where feasible. In general, this will be feasible for studies that address similar questions with respect to the population, exposure, comparison (if any), and outcomes. For data that cannot be pooled by meta-analysis, we will use the Synthesis Without Meta-analysis (SWiM) guideline checklist [20] to ensure that the quantitative narrative synthesis of data remains as free of bias, as is feasible. The checklist has 9 items that encompass critical aspects of data synthesis including grouping of studies, methods for synthesizing data, presentation of the data, and limitations of the synthesis.

### Statistical analysis

We will present the data with descriptive statistics and provide pooled estimates of outcome parameters, wherever it is feasible to perform a meta-analysis. Pooled estimates will be presented with 95% confidence intervals. Outcomes reported through dichotomous variables will be expressed as proportions and compared within and/or between groups (where applicable) using odds ratios. Outcomes reported through continuous variables will be expressed as mean (SD) and compared within and/or between groups (where applicable) using weighted mean difference. For continuous variables expressed as median (IQR), efforts will be made to convert the values to mean (SD). The default analysis will be a random effects model.

### Sensitivity analysis

A sensitivity analysis will be done by excluding the studies with high risk of bias.

### Subgroup analysis

Subgroup analysis will be conducted (where possible) based on the following characteristics:

1. Age groups: <1 year, 1–5 years, 6–12 years, and 13–18 years. The cut-off of 12 years has been chosen for one of the subgroups, because in some developing countries (including India), children till 12 years of age are managed by pediatricians, and thereafter by physicians caring for adults. Therefore, there may be literature that included children till 12 years of age only
2. Type of upper respiratory tract specimen respiratory specimen: nasopharyngeal swab/aspirate, oropharyngeal swab/aspirate, others
3. Methods for identification and comparison of microbial flora: culture versus molecular methods
4. Type of respiratory disease: infectious condition versus non-infectious condition, and acute versus chronic disease

### Assessment of heterogeneity

We will test for heterogeneity using the *I*^2^ statistic. We will interpret heterogeneity as outlined in the *Cochrane Handbook for Systematic Reviews of Interventions* [21]. An *I*^2^ statistic < 50% will be considered to be a low level of heterogeneity, 50 to 75% a moderate level, and >75% a high level. Where substantial heterogeneity is identified, we will explore possible causes for it.

### Assessment of reporting biases

Wherever possible, we will obtain the original trial protocols for comparison with the published papers to ensure that all outcomes were reported. If it is not possible to obtain the trial protocols, we will scrutinize the “Methods” section of the published paper(s) to ensure full reporting of all measured variables. We will use the Outcome Reporting Bias in Trials (ORBIT) classification system to highlight missing or incomplete outcome reporting of the outcomes [22].

If negative data were not fully reported, we will contact the primary investigators for these data. We will explore reporting bias using a funnel plot. We will also assess publication bias by looking for evidence of conference presentations not followed by subsequent journal publications.

### Risk of bias in individual studies and assessment of methodological quality

Two authors will independently assess the risk of bias and methodological limitations of each included observational study using the Newcastle–Ottawa Scale (NOS) [23]. NOS is used to assess the quality of non-randomized studies including case–control and cohort studies. The NOS contains eight items, categorized into three broad domains viz. (i) selection of the study groups, (ii) comparability of the groups, and (iii) ascertainment of either the exposure or outcome of interest for case–control or cohort studies, respectively. We will rate the quality of the studies (good, fair, and poor) by awarding stars in each domain following the guidelines of the Newcastle–Ottawa Scale. A “good” quality score will require 3 or 4 stars in selection, 1 or 2 stars in comparability, and 2 or 3 stars in outcomes. A “fair” quality score will require 2 stars in selection, 1 or 2 stars in comparability, and 2 or 3 stars in outcomes. A “poor” quality score will reflect 0 or 1 star(s) in selection, or 0 stars in comparability, or 0 or 1 star(s) in outcomes [24].

For case series and case studies, we will use the Joanna Briggs Institute (JBI) Critical Appraisal Checklist for case series [25].

We will assess the risk of bias across included studies in two ways, as per the Cochrane Handbook guidelines [21]. First, we will assess the risk of bias for an individual outcome, by making judgments about evidence quality. Second, we will try to assess the overall risk of bias across included studies by making judgments on empirical evidence of bias, likely direction of bias, and likely magnitude of bias.

### Confidence in cumulative evidence

We will present a summary of finding tables, displaying a structured summary of each review question, findings, and references to the studies contributing data to each review question.

Two reviewers will independently assess the quality of evidence of each outcome based on five GRADE considerations, i.e., study limitations, consistency of effect, imprecision, indirectness, and publication bias. We will use the methods described in the *Cochrane Handbook for Systematic Reviews of Interventions* [21], employing GRADEpro GDT software. We will justify decisions to downgrade or upgrade the quality using footnotes with comments. We will also consider the overall quality of evidence across outcomes. The quality of evidence will be rated as high, moderate, low, or very low.

## Discussion

For decades, the upper respiratory tract has been the preferred site to obtain specimens for microbiologic analysis in infants and children with various respiratory diseases including lower respiratory infections. This is despite the fact that this site is well recognized to be colonized by various microbial species almost immediately after birth. Therefore, healthy (asymptomatic) children likely carry the same micro-organisms (ncluding potentially pathogenic bacteria and viruses) in their nasopharynx, oro-pharynx, or nose that are recovered during respiratory infection. There are several reasons for this apparent paradox, notably the convenience of obtaining specimens from such sites, limited invasiveness of such procedures, and technical as well as ethical limitations in obtaining lower airway specimens, especially from the lungs. Recent data suggest that the lung itself may be home to a host of microbial species, which has not been well characterized in children.

For these reasons, it is essential to identify the spectrum of microbiota in the upper airway sites of healthy infants and children and identify any relationship(s) to organisms present in the lungs. A similar comparison in children with various respiratory diseases, especially infection, is essential. A comparison of the organisms identified from similar sites in children with and without respiratory disease will be very helpful in understanding the potential pathogenicity of organisms recovered from various sites.

This systematic review will address these knowledge gaps. We believe that this is the first comprehensive effort to systematically identify and synthesize evidence on this subject. Some of the strengths of the proposed review are the multiple review questions; prior publication of the protocol for peer review; literature search through several databases, for published as well as unpublished studies; and the robust data analysis plan.

This review also has some limitations. Our search strategy may miss sources of information available in dissertations, conference presentations, and in-house databases. The review questions cannot be addressed through randomized controlled trials of interventions. Therefore, we can include only observational studies and case series, which are fraught with various risks of bias. The anticipated heterogeneity and inability to quantitatively pool the data through meta-analysis may limit the conclusions that could be inferred. We will consider these limitations when we draw conclusions from this review.

We plan to disseminate the completed systematic review through a peer-reviewed journal publication. Data that cannot be included in the published version will be made available to anyone, on request. We expect the results of the review to be of immense benefit to pediatricians, microbiologists, nurses, and respiratory therapists, caring for children with acute and chronic respiratory diseases. It will also be useful to clinical researchers working in the fields of respiratory infection etiology, risk factors for infection, and management strategies. The findings of the review may also guide researchers undertaking primary research studies on one or more of the review questions.

## Data Availability

The dataset used or analyzed during the proposed systematic review will be available from the corresponding author on reasonable request.

## Abbreviations

JBI: Joanna Briggs Institute
MeSH: Medical Subject Headings
NOS: Newcastle–Ottawa Scale
ORBIT: Outcome Reporting Bias in Trials
PRESS: Peer Review of Electronic Search Strategies
PRISMA-P: Preferred Reporting Items for Systematic Review and Meta-Analysis- Protocol
RCT: Randomized controlled trial
SWiM: Systematic Review without Meta-analysis
